# Rosiglitazone Ameliorates Cardiac and Skeletal Muscle Dysfunction by Correction of Energetics in Huntington’s Disease

**DOI:** 10.3390/cells11172662

**Published:** 2022-08-27

**Authors:** Marta Tomczyk, Alicja Braczko, Paulina Mierzejewska, Magdalena Podlacha, Oliwia Krol, Patrycja Jablonska, Agata Jedrzejewska, Karolina Pierzynowska, Grzegorz Wegrzyn, Ewa M. Slominska, Ryszard T. Smolenski

**Affiliations:** 1 Department of Biochemistry, Medical University of Gdansk, 80-211 Gdansk, Poland; 2 Department of Molecular Biology, University of Gdansk, 80-308 Gdansk, Poland

**Keywords:** Huntington’s disease, myopathy, cardiomyopathy, rosiglitazone, molecular mechanisms, therapy, energy metabolism

## Abstract

Huntington’s disease (HD) is a rare neurodegenerative disease that is accompanied by skeletal muscle atrophy and cardiomyopathy. Tissues affected by HD (central nervous system [CNS], skeletal muscle, and heart) are known to suffer from deteriorated cellular energy metabolism that manifests already at presymptomatic stages. This work aimed to test the effects of peroxisome proliferator-activated receptor (PPAR)-γ agonist—rosiglitazone on grip strength and heart function in an experimental HD model—on R6/1 mice and to address the mechanisms. We noted that rosiglitazone treatment lead to improvement of R6/1 mice grip strength and cardiac mechanical function. It was accompanied by an enhancement of the total adenine nucleotides pool, increased glucose oxidation, changes in mitochondrial number (indicated as increased citric synthase activity), and reduction in mitochondrial complex I activity. These metabolic changes were supported by increased total antioxidant status in HD mice injected with rosiglitazone. Correction of energy deficits with rosiglitazone was further indicated by decreased accumulation of nucleotide catabolites in HD mice serum. Thus, rosiglitazone treatment may not only delay neurodegeneration but also may ameliorate cardio- and myopathy linked to HD by improvement of cellular energetics.

## 1. Introduction

Huntington’s disease (HD) is a rare neurodegenerative disease that is known to primarily affect the central nervous system. The genetic cause of HD is the occurrence of multiple repeats of the CAG nucleotide sequence within the huntingtin gene (*HTT*) localized on chromosome 4, which results in the elongation of the polyglutamine stretch in the HTT protein [1]. The elongation of the polyglutamine stretch in exon 1 *HTT* leads to the formation of insoluble huntingtin aggregates, which are observed in both the early and advanced stages of the disease [2,3]. Aggregates of the mutated form of HTT (mHTT) have been identified not only in the brain but also outside the central nervous system (CNS), e.g., in skeletal muscle [3,4,5]. Interestingly, mHTT is absent in the HD-affected heart [6,7]. It has been shown that HD patients suffer from reduced (by about 50%) muscular strength compared to healthy controls [8]. Besides skeletal muscle pathology, multiple epidemiological studies have shown that heart failure is the second leading cause of death in HD patients [9,10]. Similar findings were observed in preclinical HD models [11]. HD mice models were characterized by skeletal muscle atrophy and altered ultrastructure of transverse tubules in skeletal muscle fibers [12,13]. mHTT formation in skeletal muscle leads to defects, such as myofiber size reduction or type switching [12,14,15,16,17]. HD animal models reaffirmed cardiac pathological events noted in HD patients, such as variations in the heart rate and cardiac remodeling [7,18,19]. Moreover, heart contractile dysfunctions, which might be a part of dilated cardiomyopathy were noted [7]. Thus, nowadays HD is considered as a multisystem disorder [11,20].

HD-affected CNS and non-CNS tissues were characterized by defects in energy metabolism [21]. The striatum mitochondrial oxidative metabolism investigation underlined the selective defect of glycolysis in early and clinical symptoms in HD patients [22]. In a few independent studies of the striatum of mHTT knock-in mice, HD patients’ postmortem brains, and lymphoblasts, the adenosine-5′-triphosphate (ATP)/ adenosine-5′-diphosphate (ADP) ratio was reduced as a consequence of mHTT aggregation [23,24,25]. A decreased ATP/ADP ratio was found also in mHTT-containing striatal cells, which were linked to increased Ca^2+^ influx through N-methyl-D-aspartate (NMDA) receptors. Interestingly, the disrupted ATP/ADP ratio was normalized by blocking Ca^2+^ influx [26]. Deteriorations in energy metabolism occur also in HD-affected skeletal muscle [27]. It has been noted that the skeletal muscles of HD patients are characterized by dysfunction of oxidative metabolism [28]. Moreover, muscle ATP/phosphocreatine and inorganic phosphate levels were significantly reduced in both symptomatic and presymptomatic HD subjects [29]. Previously, we have noted that R6/2, as well as HdhQ150, two well-established HD mice models, exhibited decreased ATP, ADP, and adenosine-5′-monophosphate (AMP) concentrations in three different skeletal muscle tissues—extensor digitorum longus, tibialis anterior, and soleus. Moreover, a significant reduction of phosphocreatine (PCr) and creatine (Cr) levels and the PCr/Cr were noted [17]. Similar changes were observed in HD mice models’ hearts. We highlighted decreased concentrations of ATP and phosphocreatine as well as diminished ATP/ADP ratios [30].

Interestingly, as mentioned above, energy metabolism deterioration manifests not only in the advanced stages of the disease but also in the presymptomatic. It could be suggested that energy deficit is likely to be an early phenomenon in the cascade of events leading to HD pathogenesis. Moreover, impaired bioenergetics in HD likely represent downstream effects of both an mHTT toxic gain-of-function and an HTT loss-of-function [21]. Thus, therapeutic strategies include compounds that directly correct disrupted ATP levels in affected HD CNS as well as non-CNS tissues might be an interesting therapeutic target. Nevertheless, compounds such as the coenzyme Q10 or creatinine were widely tested and even investigated in clinical trials, but the results were not promising [31]. 

An alternative might be the application of peroxisome proliferator-activated receptors (PPARs) agonists, which have already undergone preclinical studies for the treatment of CNS, cardiovascular as well as skeletal muscle diseases. PPARs belong to the group of nuclear receptors that activate or repress target genes as heterodimers with retinoic X receptors (RxR). PPARs family included: PPAR alpha (PPARα), PPAR beta/delta (PPARβ/δ), and PPAR gamma (PPARγ) [32]. Different types of cells exhibited various expressions of PPARs; thus, the outcome of its activation might be different in various tissues [33]. In 2016, the PPAR delta receptor agonist KD3010 was tested in the HD N171-82Q mouse model. Study revealed improved motor function, reducing the progression of the neurodegenerative process, and longer survival of treated animals [34]. Nevertheless, this study was focused mainly on the evaluation of CNS function improvement. Thus, our work for the first time highlighted the effect of PPAR agonist treatment on HD mouse model grip strength, cardiac function, and HD-affected skeletal muscle and heart metabolism.

## 2. Materials and Methods

### 2.1. Animal Maintenance and Treatment

All experiments were conducted following the *Guide for the Care and Use of the Laboratory Animals* published by the European Parliament, Directive 2010/63/EU, and were approved by the local bioethical committee for the Medical University of Gdansk. Animals were maintained on a 12:12 h light-dark cycle at 25 °C, 30–40% humidity, and were provided with free access to water and a standard chow diet (Morawski, Kcynia, Poland). R6/1 (*n* = 30) aged 21 weeks old and C57BL/6J (*n* = 11) as WT mice were used in the study. R6/1 mice (*n* = 12) were treated daily for six weeks with 10 mg/kg of rosiglitazone (Sigma-Aldrich, St. Louis, MO, USA) (dissolved in 0.09% DMSO) or 0.09% DMSO (Sigma-Aldrich, St. Louis, MO, USA) administered intraperitoneally [35].

#### 2.1.1. Forelimb Grip Strength Measurement

Forelimb grip strength was measured by a grip strength meter (GSM Grip strength meter, Ugo Basile, Gemonio VA, Italy) as described earlier [36]. Briefly, the animal was held on the apparatus so that only the forelimb paws grasped the specially designed mouse flat mesh assembly. Then, the mouse was pulled back until its grip was broken, which was recorded from a digital display. The maximum values were used for analysis. Forelimb and maximal muscle strength were obtained as values of GF (gram-force) and normalized to body weights as “g/g mouse body weight.”

#### 2.1.2. Echocardiography

Echocardiographic examination was performed with a high-resolution ultrasound system (Vevo 1100, VisualSonics Inc, Toronto, Ontario, Canada) [37]. Mice were anesthetized with ketamine (Biowet Pulawy, Pulawy, Poland) (100 mg/kg) and xylazine (Biowet Pulawy, Pulawy, Poland) (10 mg/kg) intraperitoneally (i.p)., then their chest hair was removed and mice were placed on a heating pad. The probe (70 MHz) was placed over the anterior chest wall and directed to the ascending aorta in 2D mode. Then the mode was switched to Doppler flow velocity. Stroke Volume (SV), Cardiac Output (CO), Left Ventricular mass (LVmass), Ejection Fraction (EF), and Fractional shortening (FS) were recorded. 

#### 2.1.3. Analysis of Cardiac and Skeletal Muscle Glucose Usage

Analysis of cardiac and skeletal muscle glucose usage was performed within the method described before [30,38]. d-glucose-1,6-^13^C_2_ (Sigma-Aldrich, St. Louis, MO, USA) was administrated in the subcutaneous injection of a 1.8 mg/g body weight dose. Moreover, blood samples were collected from the tail vein before and after 30, 60, and 90 min of ^13^C_2_ glucose administration. Next, after animal anesthesia, heart and skeletal muscle were rapidly excised (after 90 min), and freeze clamped (after animal intubation and under artificial ventilation). 

Hearts were placed for 24 h in a freeze dryer (Modulyo, Thermo Electron Corporation, Waltham, MA, USA) at −55 °C, and then were extracted with 0.4 M perchloric acid (Sigma-Aldrich, St. Louis, MO, USA) in a 1:25 ratio, followed by neutralization with 2 M KOH (Sigma-Aldrich, St. Louis, MO, USA). Supernatants (obtained from centrifugation at 4 °C, 14,000 RPM/min for 10 min) were analyzed by LC/MS.

Blood extraction was performed using ice-cooled acetone (Sigma-Aldrich, St. Louis, MO, USA) in a 1:3 ratio. Next, samples were placed in ice for 15 min and centrifuged at 4 °C, 14,000 RPM/min for 10 min. This was followed by drying in a vacuum concentrator (JW Electronic, Warsaw, Poland) and sediments were dissolved in high-purity water (Nanopure—ultrapure water system, Barnstead, Thermo, Waltham, MA, USA) and analyzed with LC/MS. 

The heart extracts were analyzed by LC/MS using a TSQ-Vantage triple quadrupole mass detector (Thermo, Waltham, MA, USA), linked to a Surveyor chromatography system (Thermo, Waltham, MA, USA) in positive heated electrospray ionization with fragmentation mode (Tandem MS), monitoring ^13^C isotopic enrichment of fragments containing C3 of alanine or C4 of glutamate. The ^13^C glucose enrichment in blood was measured using liquid chromatography-mass spectrometry—an LCQ-Deca XP mass detector (Thermo Finnigan, San Jose, CA, USA). Fragments containing ^12^C and ^13^C glucose were detected in negative electrospray ionization with the selected ion monitoring (SIM) mode for ^12^C glucose *m*/*z* 178.00–179.40 and *m*/*z* 179.00–180.40 for d-glucose-1,6-^13^C_2_.

#### 2.1.4. Mice Tissues and Serum Collection

Tissues and serum for further analysis were collected after mice anesthesia with a ketamine/xylazine mixture (Biowet Pulawy, Pulawy, Poland) (50 mg/kg + 5 mg/kg) and artificial ventilation. Blood was collected from inferior vena cava (IVC). For serum collection, blood was centrifuged at 2000 RPM for 4 min. Mice heart and skeletal muscle were also isolated.

### 2.2. Measurement of Total Adenine Nucleotides Pool, Phosphocreatine and Creatine, and Nicotinamide Dinucleotides

Hearts and skeletal muscle (soleus) were prepared and analyzed with the high-pressure liquid chromatography (HPLC) method as previously described [30]. 

### 2.3. Investigation of Cardiac and Skeletal Muscle Mitochondrial Chain Complexes Activities

Mitochondria were isolated from the soleus muscle and heart, and prepared based on the previously described procedure [36,39]. Analysis was performed by Seahorse Metabolic Flux Analyzer (Agilent Technologies, Santa Clara, CA, USA). For electron flow experiments, isolated mitochondria were diluted in cold MAS buffer (enriched with 10 mM pyruvate (Sigma-Aldrich, St. Louis, MO, USA)2 mM malate (Sigma-Aldrich, St. Louis, MO, USA), and 4 µM FCCP (Sigma-Aldrich, St. Louis, MO, USA). A mitochondrial suspension of 25 µL was placed into Seahorse plate wells and centrifuged at 2000× *g* for 15 min at 4 °C. The concentration of mitochondrial protein was 6 µg per well. After centrifugation, 180 µL of prewarmed MAS buffer supplemented with pyruvate, malate, and FCCP was added to each well, and the plate was then placed into a non-CO_2_ incubator for 8 min. The Seahorse cartridge was filled with the following reagents: 2 µM Rotenone (Sigma-Aldrich, St. Louis, MO, USA), 2 mM succinate (Sigma-Aldrich, St. Louis, MO, USA), 4 µM Antimycin (Sigma-Aldrich, St. Louis, MO, USA), and a mix of 10 mM ascorbate (Sigma-Aldrich, St. Louis, MO, USA) and 100 µM TMPD (Sigma-Aldrich, St. Louis, MO, USA). 

### 2.4. Evaluation of Cardiac and Skeletal Muscle Citric Synthase Activity

Citric synthase activity (in soleus muscle and heart) was measured within the assay kit (Sigma-Aldrich, St. Louis, MO, USA). The activity of the enzyme is measured by following the color of 5-thio-2-nitrobenzoic acid (TNB), which is generated from 5,5′-Dithiobis-(2-nitrobenzoic acid) (DTNB) present in the reaction of citrate synthesis, and caused by the deacetylation of Acetyl-CoA. Citric synthase activity was presented as µmol/mL/min.

### 2.5. Measurement of Nucleotides Catabolites in Serum 

Mice serum was extracted with 1.3 M perchloric acid (Sigma-Aldrich, Burlington, MA, USA) (1:1 ratio). Levels of nucleotides were measured by a reverse-phase high-pressure liquid chromatography (RP-HPLC) method using the liquid chromatography (LC) system (Agilent Technologies 1100 series, Agilent Technologies Inc., Santa Clara, CA, USA), as described previously [15,30]. Results are presented as μmol/L.

### 2.6. Analysis of Total Plasma Antioxidant Status

The total antioxidant status (TAOS) in plasma was measured by the 2,2′-azino-bis(3-ethylbenzothiazoline-6-sulphonic acid (ABTS) assay, which was based on the capacity of plasma to scavenge the ABTS+ radical [40]. Briefly, the relative inhibition of ABTS+ formation, after the plasma addition, is proportional to the antioxidant capacity of the sample. For the measurement, plasma was diluted with 180 µL phosphate buffer (0.076 M NaH_2_PO_4_ (POCH, Gliwice, Poland)+ 0.23 M Na_2_HPO_4_ (Sigma-Aldrich, Burlington, MA, USA) in pure water), and then it was incubated for 10 min at room temperature in a 96-well plate with a 5 µL reaction mixture containing 7 mM ABTS (Sigma-Aldrich, Burlington, MA, USA) and 2.45 mM potassium persulfate (Sigma-Aldrich, Burlington, MA, USA) (in phosphate buffer: 0.22 M NaH_2_PO_4_ (POCH, Gliwice, Poland) + 0.37 M Na_2_HPO_4_ (Sigma-Aldrich, Burlington, MA, USA)) solved in pure water. The absorbance in the test and control samples (saline instead of plasma) was read at 630 nm. Results expressed as percentage inhibition of the reaction were calculated as follows: TAOS [%] = 100 × (Ac − At)/Ac, where Ac is the absorbance of the control sample absorbance, and At is the test sample absorbance.

### 2.7. Investigation of Serum-Free Fatty Acids and Blood Glucose Levels

The free fatty acids (FFA) concentration in serum was measured using a commercial colorimetric assay kit (Wako NEFA C test kit; Wako Chemicals, Neuss, Germany). Serum was collected after 24 h starvation. Random blood glucose levels were measured with a glucometer (Accu check Active, Roche Diabetes Care, F. Hoffmann-La Roche Ltd., Basel, Switzerland). Blood drop was collected from the tail vein of non-starved mice.

### 2.8. Statistical Analysis

Statistical significance was evaluated using Student’s t-test for comparatives of two groups. A value of *p* < 0.05 was used to denote statistical significance, and the results are expressed as mean ± SEM. All statistics were carried out using GraphPad Prism 5.00 (GraphPad Software, San Diego, CA, USA).

## 3. Results

### 3.1. Rosiglitazone Improved Grip Strength and Cardiac Function in an HD Mouse Model

Previous experimental research that investigated the cardiac and skeletal muscle function in Huntington’s disease (HD) examined those mainly other than R6/1 HD mice models (R6/2, HdhQ150, or N171-82Q). Thus, to ensure that the investigated HD mouse model exhibited any changes in skeletal muscle and cardiac functionality, we assessed the forelimb grip strength and as well as cardiac function parameters (stroke volume, ejection fraction, fractional shortening, cardiac output, and left ventricular mass) in R6/1 in comparison to healthy controls. Similar to other HD mice models, the R6/1 mice model also exhibited a reduction of forelimb grip strength as well as normalized grip strength (Supplement Figure S1). Furthermore, significant reduction in ejection fraction, fractional shortening, cardiac output as well as left ventricular mass relative to wild-type (WT) were noted (Supplement Figure S2). That results suggested the presence of serious depletion of grip strength and cardiac function. 

**Figure 1:**
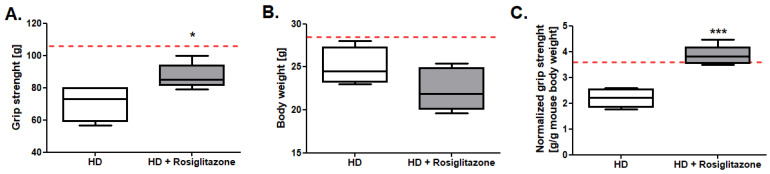
Improved grip strength in R6/1 mice treated with rosiglitazone. (**A**) Maximum forelimb grip strength. (**B**) Bodyweight (**C**) Normalized forelimb grip strength (maximum forelimb grip strength/g of body weight) in R6/1 (HD) and R6/1 with rosiglitazone treated mice (HD + Rosiglitazone). Results presented as mean ± SEM, n = 5–6, * *p* < 0.05, *** *p* < 0.001. The red dotted lines present the mean value of the investigated parameter in control, C57BL/6J mice (adapted from supplementary material data).

One of the main goals of our study was to investigate the influence of rosiglitazone on HD mouse model skeletal muscle functionality. Thus, we measured the forelimb grip strength and normalized grip strength (force normalized for mouse body weight) in R6/1 and R6/1 mice treated with rosiglitazone. We found no changes in body weight in peroxisome proliferator-activated receptor (PPAR) agonists treated mice in comparison to non-treated HD mice, while maximum, as well as normalized grip strength evaluation, indicated higher values of these parameters in HD treated with rosiglitazone (Figure 1A–C).

**Figure 2:**
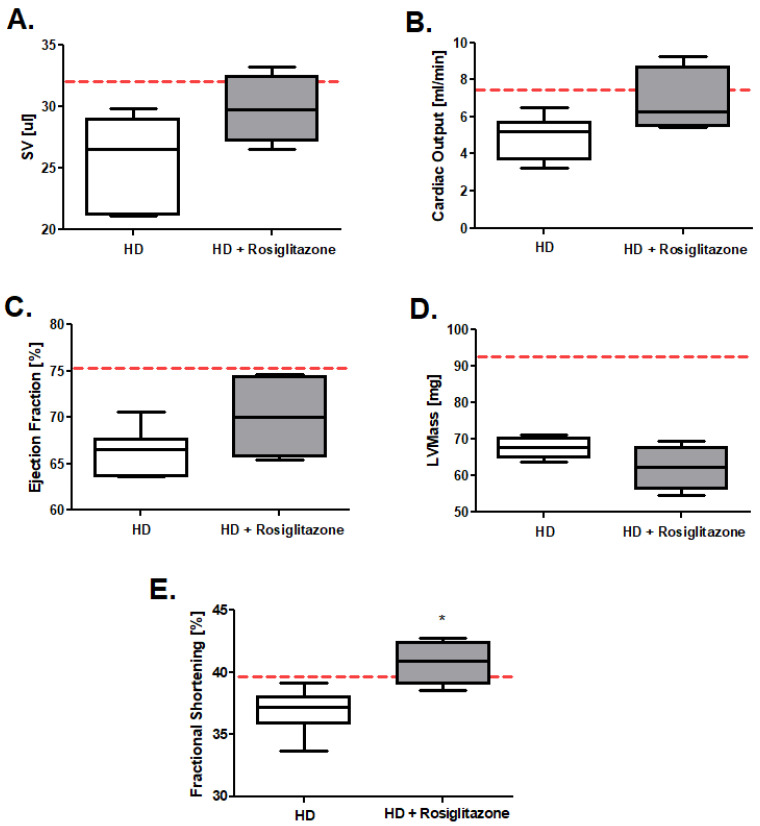
Huntington’s disease (HD) mouse model heart function improvement after rosiglitazone treatment (**A**) Stroke volume (SV), (**B**) Cardiac output, (**C**) Ejection fraction, (**D**) Left ventricle mass, (**E**) Fractional shortening in R6/1 (HD) and R6/1 with rosiglitazone treated mice (HD + Rosiglitazone). Results presented, n = 4–6, * *p* < 0.05. The red dotted lines present the mean value of the investigated parameter in control, C57BL/6J mice (adapted from supplementary material data).

We examined also the R6/1 mice’s heart function after rosiglitazone treatment (representative echocardiograms in Supplement Figure S3). We noted tendencies in the improvement of stroke volume (SV), cardiac output, and ejection fraction in HD mice treated with rosiglitazone and no changes in left ventricular mass in comparison to HD control (treated with 0.09% DMSO) (Figure 2A–D). Interestingly, we found the statistically confirmed improvement of fractional shortening in the HD mouse model injected with investigated PPAR agonist (Figure 2E).

### 3.2. Rosiglitazone Enhanced Skeletal Muscle and Cardiac Glucose Usage in an HD Mouse Model

To unravel the source of the noticed skeletal muscle as well as cardiac functionality improvement, we evaluated the in vivo glycolytic and oxidative metabolism of labeled ^13^C glucose. Metabolite tracking (4-^13^C glutamate and 3-^13^C alanine) after ^13^C glucose administration was previously extensively studied by our group [38]. Theoretical assumptions, supported by experimental studies, indicate that after ^13^C glucose administration, the heart accumulates 3-^13^C pyruvate in proportion to the fraction of glycolytic substrate, supplied by exogenous glucose relative to alternative unlabeled substrate sources (e.g., endogenous glycogen) and 4-^13^C α-ketoglutarate in proportion to the fraction of tricarboxylic acid (TCA) cycle carbon flux supported by flux through pyruvate dehydrogenase (PDH), relative to other acetyl-CoA sources (e.g., free fatty acids (FFA)). It has to be mentioned that 3-^13^C pyruvate, as well as 4-^13^C α-ketoglutarate, were present in small quantities in the heart, but occur in isotopic equilibrium with tracked 3-^13^C alanine and 4-^13^C glutamate [41]. Thus, the measurement of myocardial or skeletal muscle 3-^13^C alanine/^12^C alanine (^13^C alanine enrichment) and 4-^13^C glutamate/^12^C glutamate (^13^C glutamate enrichment) in steady-state ^13^C glucose enrichment in the blood allows for the estimation of the contribution of circulating glucose to myocardial glycolytic and oxidative flux.

**Figure 3:**
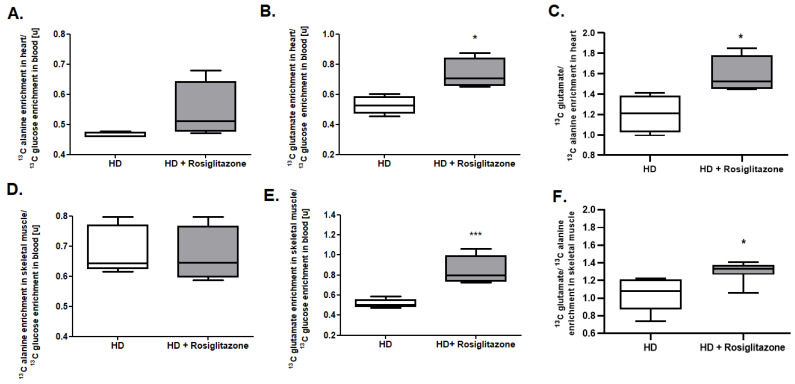
Increased glucose usage in cardiac and skeletal muscle metabolism in Huntington’s disease (HD) mouse model treated with rosiglitazone. (**A**) ^13^C alanine enrichment in heart/^13^C glucose enrichment ratio in the blood, (**B**) ^13^C glutamate enrichment in heart/^13^C glucose enrichment ratio in the blood, (**C**) ^13^C glutamate/^13^C alanine enrichment ratio in the heart, (**D**) ^13^C alanine enrichment in skeletal muscle/^13^C glucose enrichment ratio in the blood, (**E**) ^13^C glutamate enrichment in skeletal muscle/^13^C glucose enrichment ratio in the blood, (**F**) ^13^C glutamate/^13^C alanine ratio enrichment in the skeletal muscle of R6/1 (HD) and R6/1 with rosiglitazone treated mice (HD + Rosiglitazone). Data presented; n = 5; * *p* < 0.05, *** *p* < 0.001. Due to the methodological inability to compare the obtained values with previous experiments, values from control experiments (wild-type mice) are not shown. Nevertheless, the comparison of glucose usage in cardiac and skeletal muscle metabolism between control and HD mice models was already published in our two previous studies [17,30].

We observed no changes in ^13^C alanine enrichment in skeletal muscle as well as in heart to ^13^C glucose enrichment in the mouse blood ratio (Figure 3A,D). On the other hand, we noted an increased ^13^C glutamate/^13^C glucose ratio and ^13^C glutamate/^13^C alanine ratio in skeletal muscle and heart in the R6/1 mice model treated with rosiglitazone relative to HD treated with 0.09% DMSO (control), which indicates enhanced glucose oxidation as well as its overall use in cardiac and skeletal muscle metabolism (Figure 3 B,C,E,F).

Additionally, we assessed the serum-free fatty acids (FFA) as well as blood glucose concentration (Supplementary Figure S4). We found that HD mice treated with PPAR agonist were characterized by reduced blood glucose levels in comparison to non-treated mice. In the case of FFA, we found that HD mice injected with rosiglitazone do not exhibit statistically significant changes in this parameter relative to HD non-treated mice.

### 3.3. Rosiglitazone Improved Cardiac and Skeletal Muscle Adenine Nucleotides Pool

**Figure 4:**
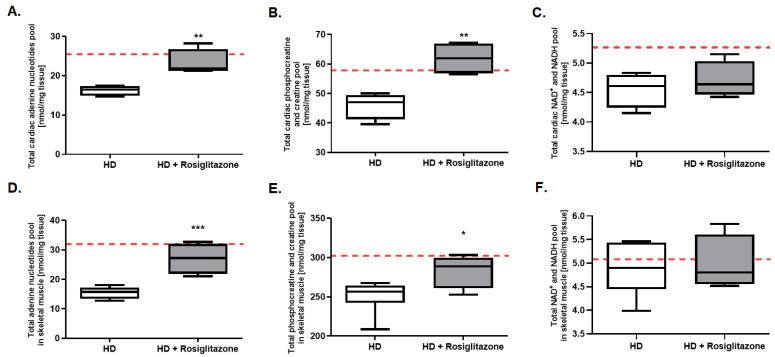
Enhanced total adenine nucleotides and total phosphocreatine creatine pools in hearts and skeletal muscles of Huntington’s disease (HD) mouse model treated with rosiglitazone. Total adenine nucleotides pool in hearts (**A**) and skeletal muscle (**D**), total phosphocreatine and creatine pool in hearts (**B**) and skeletal muscle (**E**), and total NAD+ and NADH pools in hearts (**C**) and skeletal muscle (**F**) in R6/1 (HD) and R6/1 with rosiglitazone treated mice (HD + Rosiglitazone). Results presented as mean ± SEM, n = 4–6, * *p* < 0.05, ** *p* < 0.01, *** *p* < 0.001. The red dotted lines present the mean value of the investigated parameter in control, C57BL/6J mice (adapted from our previous works [17,42]).

Further analysis tested whether rosiglitazone may affect overall skeletal muscle and cardiac energy metabolism. While accurate quantitative analysis of unstable metabolites such as adenosine-5′-triphosphate (ATP) and phosphocreatine was not possible due to limitations of the tissue collection procedure we were able to collect data on total pools of relevant metabolites. We noted that total cardiac and skeletal muscle adenine nucleotides pools were elevated in rosiglitazone-injected mice (Figure 4A,D). Similar increases were found in total creatine pools (Figure 4B,E). On the other hand, there were no changes in the total cardiac and skeletal muscle NAD^+^ and NADH pool (Figure 4C,F).

### 3.4. Rosiglitazone Led to Cardiac and Skeletal Muscle Mitochondria Functionality Changes

The initial step of this part of our research was the examination of oxygen consumption rate (OCR) in coupled and un-coupled cardiac and soleus mitochondria of R6/1 (HD) as well as control WT mice. Moreover, we assessed the activities of mitochondrial complex I (with pyruvate, malate, and FCCP), II (after rotenone and succinate addition), and complex IV (after addition of TMPD and ascorbate) in isolated mitochondria. 

We did not observe differences in OCR between coupled cardiac and soleus muscle mitochondria isolated from HD and WT mice at any measurement points (Supplementary Figure S5A,C). The level of respiration in isolated cardiac mitochondria in state 2, state 3 ADP, state 4o, and state 3u were similar in both strains (Supplementary Figure S5B). On the other hand, examination of mitochondrial respiration states in soleus muscle highlighted the reduced state 3u (controlled exclusively by substrate oxidation, its reduction might suggest dysfunction in respiratory chain components, substrates translocases, or dehydrogenases) (Supplementary Figure S5D). Interestingly, evaluation of R6/1 mice cardiac and soleus muscle mitochondrial OCR and complexes activities in an uncoupled state revealed complex I hyper-activation in comparison to WT (Supplementary Figures S6A,B and S7A,B). No changes in cardiac and skeletal muscle mitochondrial complex IV respiration were found between HD and control mice (Supplementary Figures S6D and S7D). Nevertheless, some difference was found between those investigated tissues. Indeed, R6/1 mice heart exhibited increased mitochondrial complex II respiration (Supplementary Figure S6A,C), while soleus muscle exhibited opposite reduced tendency (Supplementary Figure S7A,C).

Next, we evaluated the activity of citric synthase (CS, the indicator of mitochondria amount) and reactive oxygen species levels (ROS, the indicator of oxidative stress) in the hearts and soleus of HD and control mice. We found no changes in CS activity and increased ROS levels in R6/1 mice’s hearts and skeletal muscle in comparison to control mice (Supplementary Figure S8A–D). Results ensure the presence of mitochondrial functionality changes in HD-affected heart and skeletal muscle in investigated HD mice model.

**Figure 5:**
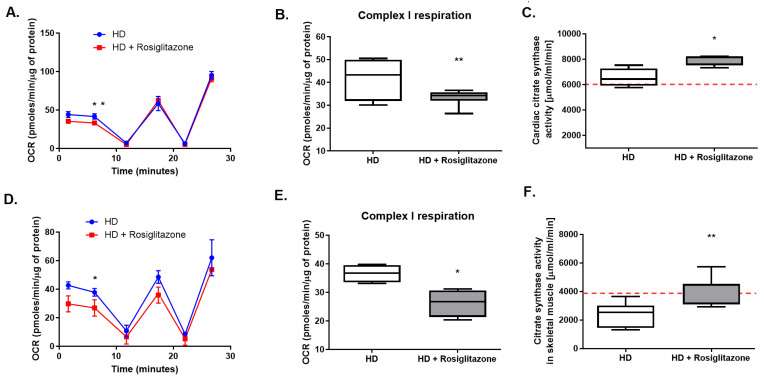
Diminished complex I respiration in mitochondria as well as increased cardiac and skeletal muscle synthase activity in R6/1 mice treated with rosiglitazone. OCR data from isolated cardiac (**A**) and soleus muscle (**D**), complex I respiration in cardiac (**B**) and soleus muscle (**E**) mitochondria, and cardiac (**C**) and skeletal muscle (**F**) citrate synthase activity in R6/1 (HD) and R6/1 with rosiglitazone treated mice (HD + Rosiglitazone). Data presented as mean ± SEM, *n* = 4–6, * *p* < 0.05, ** *p* < 0.01. The red dotted lines present the mean value of the investigated parameter in control, C57BL/6J mice (adapted from supplementary materials). Due to the methodological inability to compare the obtained values with values from other experiments conducted on Seahorse metabolic analyzer, values from control experiments (wild-type mice) are not shown. Nevertheless, the comparison of the OCR values between control and HD mice models is present in the supplementary materials.

It is well known that rosiglitazone may improve mitochondrial number and metabolism. Thus, we aimed to evaluate the mitochondrial oxidative chain complexes’ respiration and activities in hearts and mitochondria-rich, red soleus muscle, isolated from HD mice treated with rosiglitazone as well as non-treated HD mice. There were no changes in complex II and complex IV respiration, while reduced complex I respiration in hearts and soleus of R6/1 treated with PPAR agonist (HD + Rosiglitazone) relative to R6/1 (HD) were noted (Figure 5A,B,D,E). 

To investigate the effect of rosiglitazone on mitochondrial biogenesis in HD-affected tissues (heart and skeletal muscle), we assessed the activity of citric synthase. Interestingly, we noted elevated activity of this enzyme in investigated tissues isolated from HD mice treated with rosiglitazone in comparison to non-treated HD mice (Figure 5C,F). 

### 3.5. Rosiglitazone Abolished Changes in Energy Deficits Biomarkers and Improved Total Antioxidant Status in HD Mouse Model Serum

**Figure 6:**
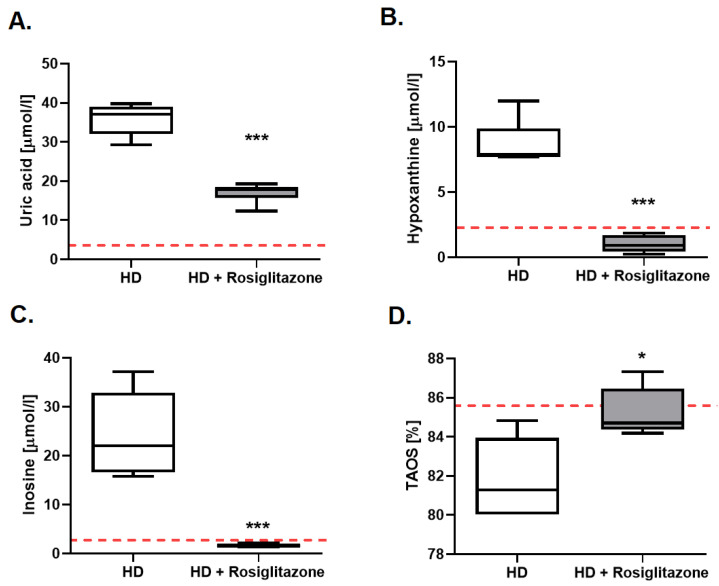
The reduced serum concentration of purine catabolites and improved total plasma antioxidant status (TAOS) in R6/1 mice treated with rosiglitazone. Serum uric acid (**A**), hypoxanthine (**B**), inosine (**C**) concentration, and total plasma antioxidant status (**D**) in R6/1 (HD) and R6/1 with rosiglitazone treated mice (HD + Rosiglitazone). Data presented as mean ± SEM, *n* = 5–6, * *p* < 0.05, *** *p* < 0.001. The red dotted lines present the mean value of the investigated parameter in control, C57BL/6J mice (adapted from supplementary materials and our previous work [30]).

Improvement of energy metabolism may lead to changes in nucleotides’ catabolite profile in serum. Thus, the next step of our research was the evaluation of the concentration of uric acid, hypoxanthine, and inosine in the serum of R6/1 mice treated with rosiglitazone. We found reduced levels of all investigated nucleotide catabolites in HD mice injected with PPAR agonist in comparison to non-treated mice (Figure 6A–C).

Due to the earlier noted increase of ROS in HD mice hearts and skeletal muscles, we also investigated total plasma antioxidant status (TAOS) in R6/1 and wild-type littermates. We established a reduced value of this parameter in HD mice (Supplementary Figure S9). Following these findings, we aimed to assess TAOS in HD mice treated with rosiglitazone in comparison to the non-treated group, and noted enhancement of its value in mice injected with PPAR agonist (Figure 6D). 

## 4. Discussion

**Figure 7:**
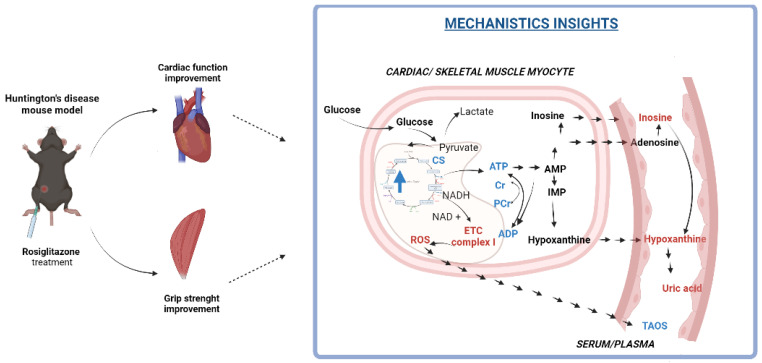
A model depicting the mechanism by which rosiglitazone may lead to improvement of cardiac and skeletal muscle functionality in the R6/1 (Huntington’s disease) mouse model. Rosiglitazone led to cardiac and skeletal muscle: 1. Mitochondrial number enhancement (measured by citric synthase activity), 2. Increased glucose oxidation and its use in overall metabolism (measured by ^13^C glutamate/^13^C glucose as well as ^13^C glutamate/^13^C alanine enrichment) and 3. Changes in mitochondrial functionality (diminished mitochondrial complex I activity). Those factors may lead to improvement of cardiac and skeletal muscle energy metabolism (enhancement of total adenine nucleotides and total phosphocreatine and creatine levels) and may contribute to diminished serum concentration of adenine nucleotides catabolites. Changes in mitochondria functionality may also contribute to improvement of total plasma antioxidant status.Red color: downregulation, blue color: upregulation. Created with Bioreder.com on 7 April 2022.

This study highlighted that the rosiglitazone treatment improves grip strength and cardiac function in Huntington’s disease (HD) mouse model R6/1 (Figure 7). These functional changes were accompanied by enhanced total adenine nucleotide and total creatine pools, increased glucose oxidation, changes in mitochondrial number (indicated as increased citric synthase activity), and reduction of mitochondrial complex I activity. Correction of energy deficits with rosiglitazone abolished, as noted in our previous research, the accumulation of nucleotide catabolites in HD mice serum [30]. Moreover, enhancement of energy metabolism and changes in mitochondrial complex I lead to improvement of oxidative balance highlighted as an increased total antioxidant status in HD mice injected with rosiglitazone.

Rosiglitazone is a synthetic agonist of peroxisome proliferator-activated receptor (PPAR)-γ, which is commonly used to reverse insulin resistance in patients with type II diabetes [43]. Interestingly, it has also been tested as a neuroprotective agent in HD, where it significantly attenuated toxicity induced by mutant huntingtin (mHTT) in striatal cells [44]. Rosiglitazone treatment also significantly reduced mHTT aggregates in the mHTT expressing neuroblastoma cell line [45]. Moreover, administration of rosiglitazone significantly improved motor function, rescued brain-derived neurotrophic factor (BDNF) deficiency in the cerebral cortex, and prevented loss of orexin-A-immunopositive neurons in the hypothalamus of N171-82Q HD mice [35]. Similar results were also noted in the HD rat model (injected with quinolinic acid) treated with rosiglitazone [46]. However, our study for the first time indicated that PPAR-γ agonist treatment of the HD mouse model also improved skeletal muscle as well as heart functionality. Although PPAR-γ expression in skeletal muscle and the heart is relatively small, there are studies indicating that these receptors may play an important role in its metabolism and function [47,48]. Treating fibromyalgia (multisystem failure process involving the immune, musculoskeletal, and central nervous system ) in rats with PPAR-γ agonist, pioglitazone resulted in a significant improvement of skeletal muscle functions, reduced fatigability, and rapid recovery from fatigue [49], which is consistent with our results. Blocking the PPAR-γ pathway, though administration of GW9662, counteracted pioglitazone’s protective effects [49]. Interestingly, experimental studies have also shown a reduction in the level of peroxisome proliferator-activated receptor-gamma coactivator (PGC-1α), one of the proteins activated by PPAR-γ in skeletal muscles of HD mice models as well as HD patients [50]. Additionally, the pharmacological activation of this co-activator led to the increased expression of skeletal muscle fiber proteins that suggested an important role of the PPAR pathway in the development of HD-related myopathy [51]. Moreover, rosiglitazone treatment seems to have an impact also on the cardiovascular system [43]. Consistently with our observation, rosiglitazone administration led to improvement of cardiac function (contractile dysfunction and the protection of myocardial injury during ischemic/reperfusion) in different animal models [52,53,54]. Nevertheless, opposite findings have also been reported. Growing evidence has demonstrated adverse effects of rosiglitazone, including increased risk of acute myocardial infarction and heart failure, which was one of the causes of its withdrawal from EU countries [55,56]. Besides its controversy, rosiglitazone seems to be an interesting therapeutic tool in HD due to unique metabolic alterations reported in this disease.

Indeed, our previous research indicated that HD cardio- and myopathy were linked with deficits in energy metabolism [17,30]. We noted that HD mice models exhibited decreased glucose oxidation in skeletal muscle and heart. This change may reduce production capacity for adenosine-5′-triphosphate (ATP), especially if combined with reduced oxygen supply. Glucose is a better substrate for energy supply ensuring cell survival. Its oxidation generates more ATP molecules concerning oxygen consumption than other metabolic fuels. Thus, increasing glucose oxidation in HD-affected skeletal muscle and heart may result in an improvement of its function. PPAR agonists could induce such an effect. Indeed, How et al. noted that rosiglitazone treatment diminished cardiac fatty acids and increased cardiac glucose oxidation in diabetic mice [57]. Rosiglitazone enhanced also glucose oxidation, and thus its overall use in metabolism in our study. Similar to our results, it was accompanied by reduced blood glucose levels caused by increased whole-body glucose uptake and its reduced hepatic release [57,58]. One may conclude that rosiglitazone treatment may help cardiac and skeletal muscle function in HD mice by treating impaired glucose homeostasis that may develop at later stages of the disease instead of directly targeting primary HD defects [59]. Although R6/1 displayed some signs of impaired glucose tolerance (including abnormal glucose handling and higher glucose plasma and insulin levels in the glucose challenge), it did not manifest as diabetes due to normal peripheral insulin sensitivity. Moreover, the fasting plasma glucose levels were similar to the values in wild-type mice, as described by different investigators; thus, this experimental model is recognized as a nondiabetic HD mouse model [59,60,61]. Interestingly, the glucose concentration reduction after rosiglitazone was also observed in non-diabetic individuals [58]. This suggests that rosiglitazone treatment could be beneficial for skeletal muscle and cardiac metabolism in HD in a way that is independent of diabetic status.

As mentioned earlier, PPAR-γ is not highly expressed in cardiac and skeletal muscle tissue, and so the effect of rosiglitazone on substrate metabolism might be most likely indirect to the changes in cardiac substrate supply, rather than a direct effect of PPARγ on cardiac or skeletal muscle. Nevertheless, our study indicated that this metabolic shift resulted in improvement of total adenine nucleotides as well as total phosphocreatine and creatine pool in R6/1 mice model skeletal muscle and heart. Moreover, energy deficits improved via rosiglitazone abolished also the massive elevation in nucleotides catabolites (inosine, hypoxanthine, and uric acid) concentration in HD mouse serum observed in our previous research [30]. Indeed, we found an increase in inosine, hypoxanthine, xanthine, and uric acid in the sera of the HD murine models. More importantly, we found also that hypoxanthine levels were elevated also in the plasma of symptomatic HD patients and correlated with HD progression parameters [30]. We suggested earlier that catabolites that we observed in sera were released by the affected heart and/or skeletal muscle tissues, which is in the line with the current study. 

Changes in energy metabolism in HD-related myopathies were linked with disruption in mitochondrial structure [50,62,63]. It has been reported that rosiglitazone treatment increases mitochondrial biogenesis in the brain and others mice tissues [44,64,65]. Indeed, we also found the increased activity of citrate synthase, a commonly used marker of mitochondrial abundance, in HD mouse model skeletal muscle and hearts treated with rosiglitazone [66]. Mitochondrial dysfunction in HD was linked also to its functionality changes including disruption of mitochondrial metabolism, calcium overload, and thus oxidative stress induction [21,67,68,69,70]. It has been shown that mHTT intracellular aggregation leads to increased reactive oxygen species (ROS) production [71]. ROS play also an important role in skeletal muscle atrophy and heart failure [72,73,74]. Abnormalities of superoxide dismutase activity and glutathione peroxidase, antioxidant enzymes involved in ROS breakdown, were also found in the HD-affected cardiac mitochondria [75]. Interestingly, we noted also an increased ROS level in R6/1 mice’s hearts and skeletal muscle. 

It is well known that mitochondrial complex I and III, but especially complex I, are considered to be the main sites of ROS production [76]. However, data that highlighted mitochondrial complexes activity in HD-affected skeletal muscle and heart are ambiguous. Indeed, no alterations of the mitochondrial electron transport chain activity were found in the skeletal muscle of 12-weeks-old R6/2 mice or skeletal muscle of 15-months-old HdhQ111 knock-in mice [77,78]. On the other hand, other analyses, performed on muscles of R6/2 mice at late stages of disease progression, reported a significant reduction of the activity of the complex IV or both, complex I and IV [6,79]. Our current study underlined the opposite tendency. We found that mitochondria of the soleus of the R6/1 mice model, despite reduced complex II and no changes in IV activity, exhibited elevated complex I activity. A similar tendency in complex I respiration we found in the mitochondria isolated from R6/1 mice hearts. Moreover, in contrast to mitochondria isolated from skeletal muscle the improvement of complex II respiration was noted. While Kojer et al. highlighted no changes in mitochondrial oxidative chain complexes in R6/2 mice hearts [6]. We suggested that such complex I hyperactivation may be the cause of observed increased ROS production and thus HD cardiac and skeletal muscle mitochondria dysfunction. Nevertheless, more mechanistic studies on isolated mitochondria or cardiac and muscle cells are needed to clarify the origin of this activation. 

There is a study indicating that rosiglitazone treatment rapidly decreases the activities of mitochondrial respiratory complex I and III without modifying complex II in liver mitochondria, and in this way diminished intracellular ROS production [80]. Rabol et al. showed that skeletal muscle mitochondria isolated from patients with diabetes type 2 supplemented with rosiglitazone for 12 weeks also exerted an inhibitory effect on complex I [81]. It is in the line with our data, highlighting that rosiglitazone treatment of HD mouse model leads to reduction of complex I activity in mitochondria isolated from skeletal muscle and heart. That may suppress the ROS overproduction and improve cardiac and skeletal muscle functionality. Indeed we also found the improvement of total plasma antioxidant status (TAOS), one of the oxidative balance indicators in rosiglitazone-treated mice, which suggested its correction [82]. It is known that the evaluation of this parameter was applied to reveal oxidative balance instead of measuring different oxidant and antioxidant molecules individually [83,84]. We noted also the reduction in TAOS in R6/1 relative to control mice. It supports the paradigms mentioned above regarding the presence of oxidative stress in HD mouse models. Interestingly, recently Yildiz et al. also underlined a significant reduction in TAOS in patients with Alzheimer’s disease and other CNS diseases [85]. Unfortunately, no data were available from HD patients.

## 5. Conclusions and Perspectives

Several earlier studies highlighted the usefulness of anti-diabetic drugs (primarily peroxisome proliferator-activated receptors (PPARs) agonists, like rosiglitazone) in the treatment of patients with other neurodegenerations such as Alzheimer’s disease [86]. Hervas et al. (2017) noted that administration of metformin—another anti-diabetic drug that stimulates adenosine monophosphate-activated protein kinase (AMPK)—was associated with better cognitive function and motor function improvement in Huntington’s disease (HD) patients [87]. Although, usage of an AMPK activator in HD might be beneficial only in the first stages of the disease where AMPK is downregulated, not in advanced stages where AMPK is already upregulated in HD-affected tissues (brain as well as skeletal muscles and heart) [30,87,88,89]. PPAR agonists could be more effective since PPAR and its signaling are downregulated in all HD stages [90].

Despite numerous basic studies highlighting the role of PPAR agonists in HD, more clinical studies are needed to clarify its possible usage in HD treatment. These studies should especially identify whether non-diabetic or diabetic HD patients would benefit more, and identify side effects. Our research conducted on non-diabetic R6/1 HD mouse models indicated that even in non-diabetic HD patients, such treatment could be considered.

## 6. Limitations

It has to be mentioned that grip strength evaluated in this study does not selectively identify a change in skeletal muscle function. Besides skeletal muscle functionality, the central nervous system (CNS), motor neurons, and neuromuscular transmission also contribute to overall grip strength. Thus, enhancement of this parameter after peroxisome proliferator-activated receptors (PPARs) agonist treatment might be related to the improvement of the CNS and its signaling (peripheral nerve functionality enhancement), while cardiac function recovery after rosiglitazone therapy might be linked with the improvement of the heart–brain axis. Nonetheless, dysfunction of peripheral cells was noted also in vitro studies, suggesting that the pathogenesis of Huntington’s disease (HD) in the heart and skeletal muscle may be independent of the CNS [1,91]. 

It is well known that skeletal muscle can continue to generate force at low adenosine-5′-triphosphate (ATP) levels. Indeed, it has been shown that maximum skeletal muscle force does not decrease until the concentration of ATP is less than 20 µM [92]. Moreover, skeletal muscle force was shown to increase by 10% in 0.5–1 mM of ATP concentration [93,94]. Changes in ATP levels might also affect muscle function by altering the SR Ca2+ ATPase (SERCA pump) and excitation-contraction coupling [95]. Interestingly, there is work showing altered excitation-contraction coupling in HD-affected muscle [96,97]. 

Furthermore, maximal skeletal muscle force occurs when blood flow is blocked to the muscle due to the contraction. These adaptations gave the ability of a skeletal muscle to continue its function in extreme metabolic conditions. Thus, as observed in our research, improved skeletal muscle glucose metabolism and total adenine nucleotides pool after rosiglitazone treatment may not result in improved skeletal muscle contractility parameters. On the other hand, our previous research indicated that the skeletal muscle of HD mice models was characterized by progressive impairment of the contractile and significant loss of motor units, accompanied by diminished skeletal muscle glucose oxidation and deterioration in energy metabolism [17]. Thus, one may conclude that improvement of these parameters in HD-affected skeletal muscle may have a positive impact on its functionality; however, more experimental studies are needed to clarify this assumption.

Despite these limitations, our study for the first time highlighted that the treatment of the HD mouse model with PPAR-γ agonists like rosiglitazone induces alterations in skeletal muscle and heart metabolism that may contribute to enhanced grip strength and improvement of cardiac function. Thus, these molecules might be an interesting therapeutic tool to treat not only neurodegeneration but also cardiomyopathy and myopathy linked to HD.

## Data Availability

Not applicable.

## Supplementary Materials

The following supporting information can be downloaded at: https://www.mdpi.com/article/10.3390/cells11172662/s1, Figure S1: Reduced grip strength in R6/1 mice in comparison to healthy controls (C57BL/6J), Figure S2: Deterioration of heart function in R6/1 mice model, Figure S3: Representative echocardiograms from R6/1 mice model (A.) and R6/1 mice model treated with rosiglitazone (B.), Figure S4: Blood glucose and serum-free fatty acids concentration in R6/1 mice (HD) as well as HD with Rosiglitazone (HD+ Rosiglitazone), Figure S5: Representative Seahorse XF assays on coupled cardiac and skeletal muscle mitochondria isolated from HD and control mice, Figure S6: Representative Seahorse XF assays on isolated cardiac mitochondria of HD and control mice, Figure S7: Representative Seahorse XF assays on isolated soleus muscle mitochondria of HD and control mice., Figure S8: Mitochondrial functionality parameters (citrate synthase and ROS) in hearts and skeletal muscle of HD and control mice, Figure S9: Total plasma antioxidant status in C57Bl (control) and R6/1 (HD) mice.
