# Supplementary material for: Rosiglitazone Ameliorates Cardiac and Skeletal Muscle Dysfunction by Correction of Energetics in Huntington’s Disease
Source: Cells. 2022 Aug 27;11(17):2662. doi: 10.3390/cells11172662 (PMC9454785; doi:10.3390/cells11172662)
Supplement: Supplementary file 1 [file cells-11-02662-s001.zip › cells-1872827-SI.pdf]

Supplementary Materials

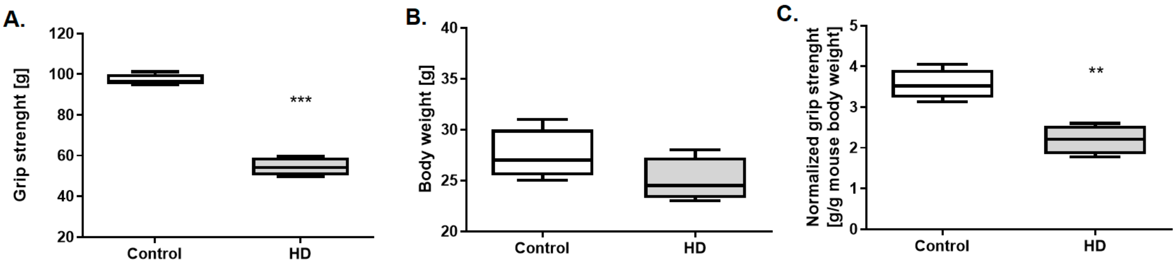

Figure S1. Reduced grip strength in R6/1 mice in comparison to healthy controls (C57BL/6J). (**A**) Maximum fore limb grip strength. (**B**) Body weight (**C**) Normalized fore limb grip strength (maximum forelimb grip strength/ g of body weight) in control and R6/1 mice (HD). Results presented as as mean ± SEM, n = 5-6, **$p < 0.01$, ***$p < 0.001$.

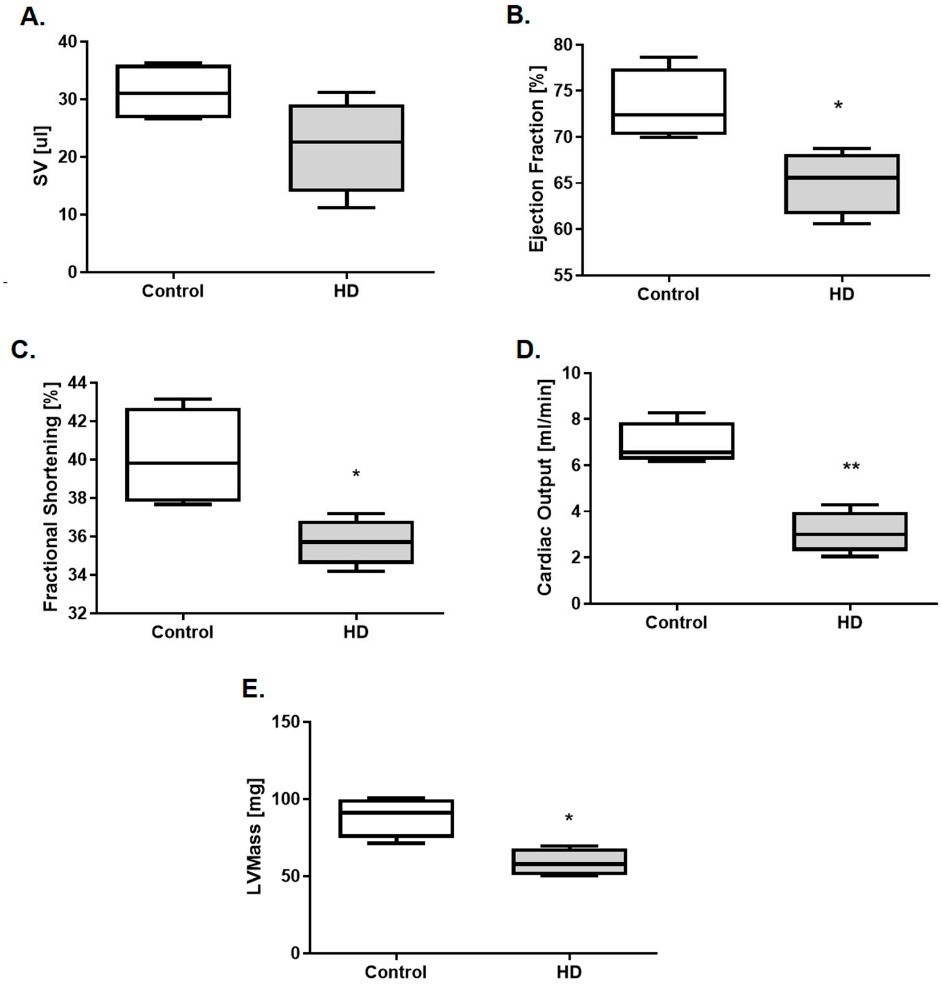

Figure S2. Deterioration of heart function in R6/1 mice model. (**A**) Stroke volume (SV), (**B**) Cardiac output, (**C**) Ejection fraction, (**D**) Left ventricle mass (**E**) Fractional shortening in wild-type (control) and R6/1 mice (HD). Results presented as mean ± SEM, n = 5, *$p < 0.05$, **$p < 0.01$.

A.

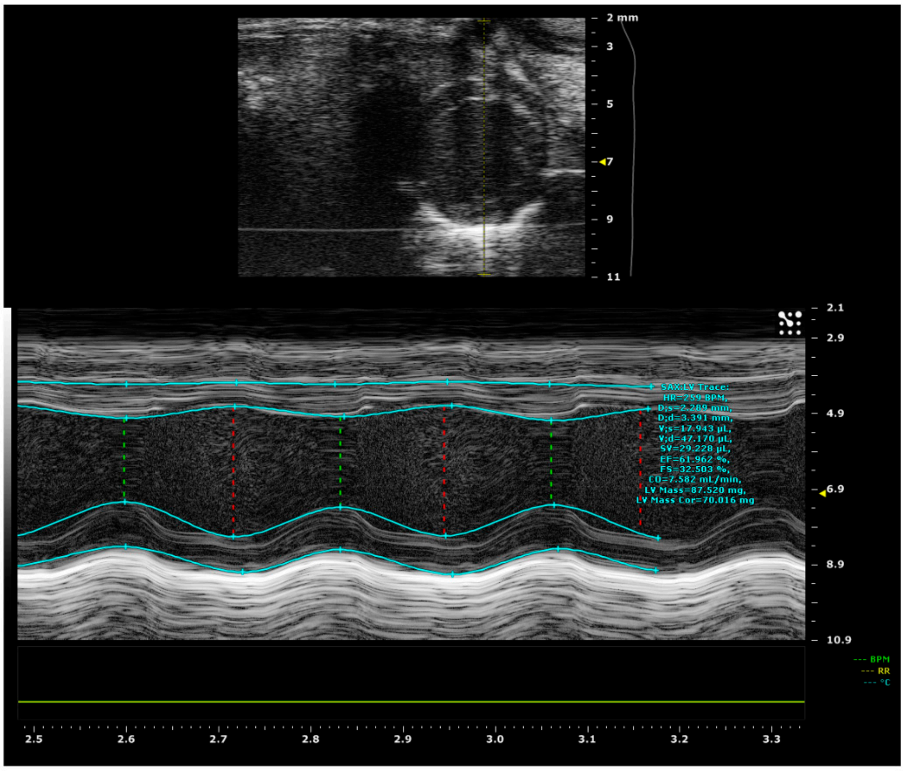

B.

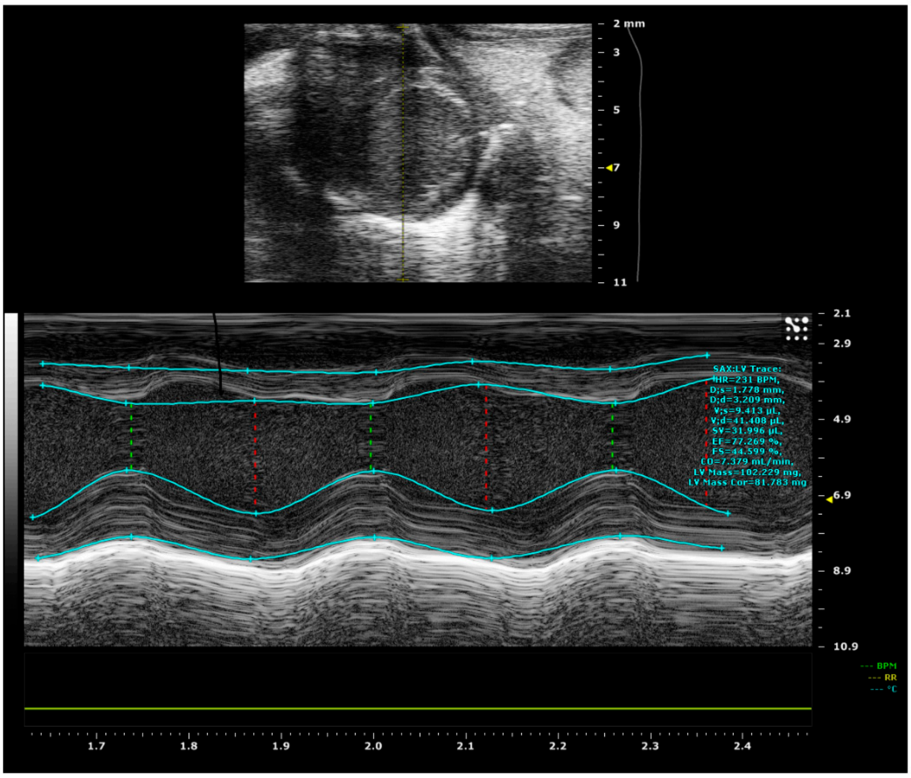

Figure S3. Representative echocardiograms from R6/1 mice model (**A**) and R6/1 mice model treated with rosiglitazone (**B**).

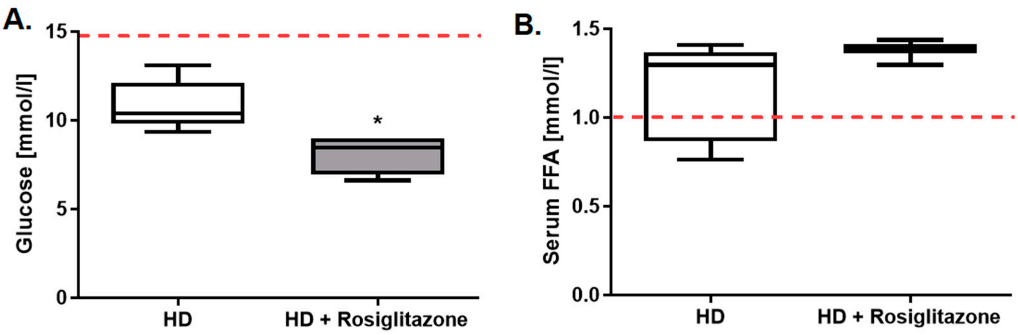

Figure S4. Blood glucose (**A**) and serum free fatty acids (**B**) concentration in R6/1 mice (HD) as well as HD with Rosiglitazone in 10mg/kg body weight dose treatment (HD+ Rosiglitazone). Data presented as mean ± SEM, n= 6, * p<0.05. The red dotted lines present the mean value of the investigated parameter in control, C57BL/6J mice (adapted from our previous work:[1]

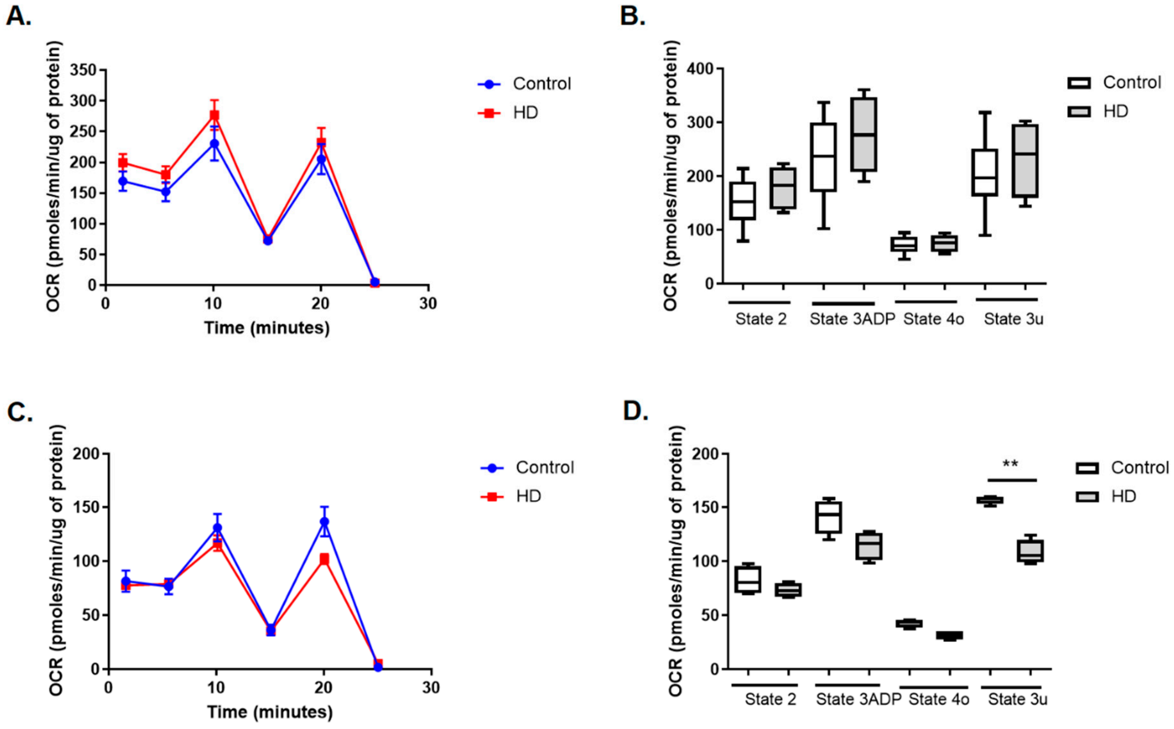

Figure S5. Representative Seahorse XF assays on coupled cardiac and skeletal muscle mitochondria isolated from HD and control mice. Graphical presentation of Coupling Assay performed on cardiac (**A**) and soleus muscle (**C**) mitochondria, and comparison of individual respiration states in cardiac (**B**) and skeletal muscle (**D**) mitochondria of C57Bl/6J (control) and R6/1 (HD) mice. Data presented as as mean ± SEM, n = 4, ** $p < 0.01$.

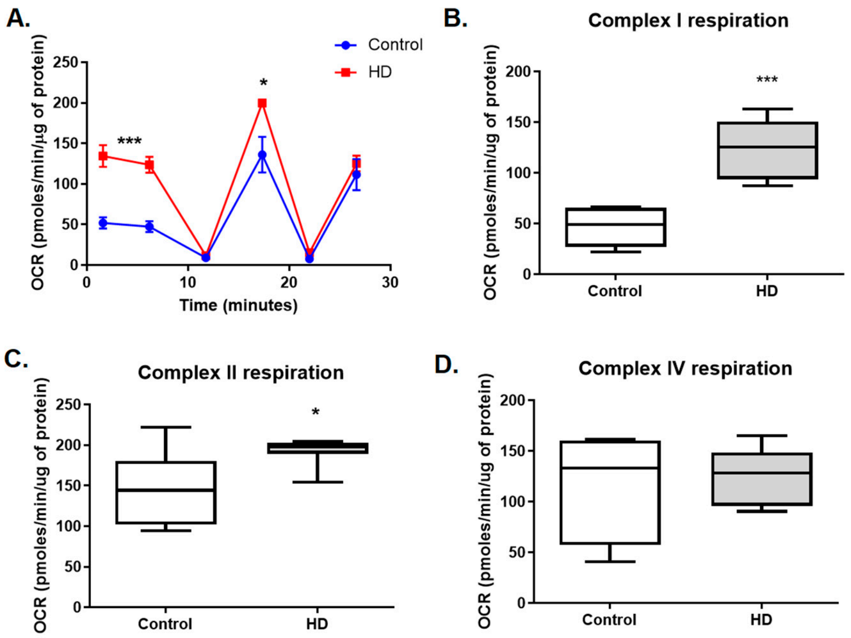

Figure S6. Representative Seahorse XF assays on isolated cardiac mitochondria of HD and control mice. (**A**) An illustrative example of mitochondrial electron flow assay performed on uncoupled cardiac mitochondria. (**B**) Complex I respiration, (**C**) Complex II respiration, (**D**) Complex IV respiration in C57Bl (control) and R6/1 (HD) mice. Data presented as mean ± SEM, n = 4–6, * $p < 0.05$, ***$p < 0.001$.

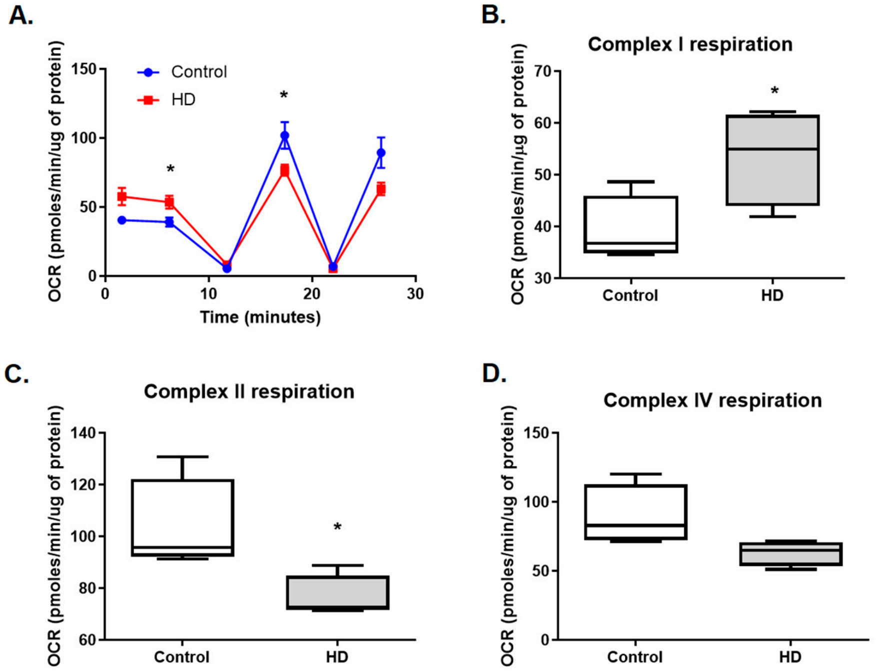

Figure S7. Representative Seahorse XF assays on isolated soleus muscle mitochondria of HD and control mice. (**A**) An illustrative example of mitochondrial electron flow assay performed on uncoupled soleus muscle mitochondria. (**B**) Complex I respiration, (**C**) Complex II respiration, (**D**) Complex IV respiration in C57Bl (control) and R6/1 (HD) mice. Data presented as mean ± SEM, n = 4-6, * $p < 0.05$.

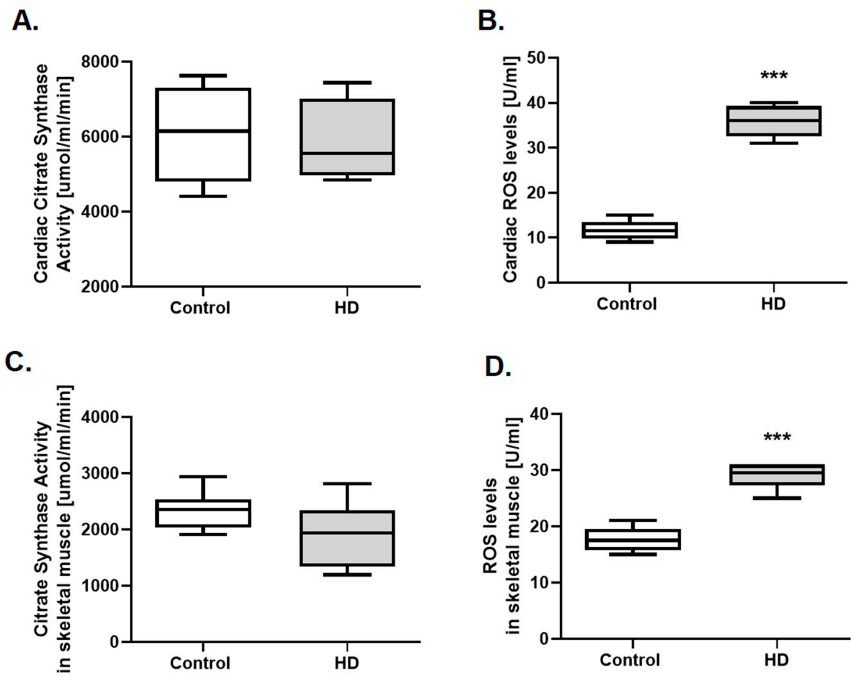

Figure S8. Mitochondrial functionality parameters (citrate synthase and ROS) in hearts and skeletal muscle of HD and control mice. Citrate synthase activity in the heart (**A**) and soleus muscle (**C**), and Levels of reactive oxygen species (ROS) in hearts (**B**) and skeletal muscle (**D**) of C57Bl (control) and R6/1 (HD) mice. Data presented as mean ± SEM, n = 4-6,****p* < 0.001.

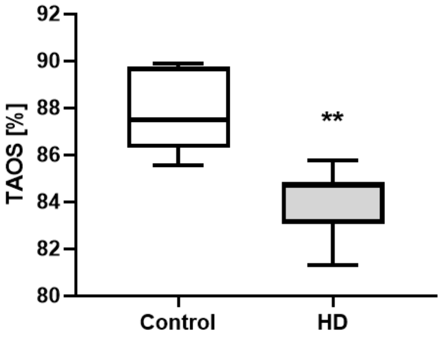

Figure S9. Total plasma antioxidant status in C57Bl (control) and R6/1 (HD) mice. Data presented as mean ± SEM, n = 5, ***p* < 0.01.

Reference

1. Tomczyk, M.; Braczko, A.; Jablonska, P.; Mika, A.; Przyborowski, K.; Jedrzejewska, A.; Krol, O.; Kus, F.; Sledzinski, T.; Chlopicki, S.; et al. Enhanced Muscle Strength in Dyslipidemic Mice and Its Relation to Increased Capacity for Fatty Acid Oxidation. *Int. J. Mol. Sci.* **2021**, *22*, 12251. https://doi.org/10.3390/ijms222212251.
